# Nerve Regenerative Effects of GABA-B Ligands in a Model of Neuropathic Pain

**DOI:** 10.1155/2014/368678

**Published:** 2014-07-15

**Authors:** Valerio Magnaghi, Luca Franco Castelnovo, Alessandro Faroni, Erica Cavalli, Lucia Caffino, Alessandra Colciago, Patrizia Procacci, Giorgio Pajardi

**Affiliations:** ^1^Dip. Scienze Farmacologiche e Biomolecolari, Università degli Studi di Milano, Via G. Balzaretti 9, 20133 Milan, Italy; ^2^Blond McIndoe Laboratories, Institute of Inflammation and Repair, Faculty of Life Science, The University of Manchester, 3.107 Stopford Building, Manchester M13 9PT, UK; ^3^Hand Surgery Department, St. Joseph MultiMedica Hospital of Milan, Via San Vittore 12, 20123 Milan, Italy; ^4^Dip. Scienze Biomediche per la Salute, Università degli Studi di Milano, Via L. Mangiagalli 31, 20133 Milan, Italy; ^5^Dip. Scienze Cliniche e di Comunità, Plastic Surgery School, Università degli Studi di Milano, Via San Barnaba 22, 20122 Milan, Italy

## Abstract

Neuropathic pain arises as a direct consequence of a lesion or disease affecting the peripheral somatosensory system. It may be associated with allodynia and increased pain sensitivity. Few studies correlated neuropathic pain with nerve morphology and myelin proteins expression. Our aim was to test if neuropathic pain is related to nerve degeneration, speculating whether the modulation of peripheral GABA-B receptors may promote nerve regeneration and decrease neuropathic pain. We used the partial sciatic ligation- (PSL-) induced neuropathic model. The biochemical, morphological, and behavioural outcomes of sciatic nerve were analysed following GABA-B ligands treatments. Simultaneous 7-days coadministration of baclofen (10 mg/kg) and CGP56433 (3 mg/kg) alters tactile hypersensitivity. Concomitantly, specific changes of peripheral nerve morphology, nerve structure, and myelin proteins (P0 and PMP22) expression were observed. Nerve macrophage recruitment decreased and step coordination was improved. The PSL-induced changes in nociception correlate with altered nerve morphology and myelin protein expression. Peripheral synergic effects, via GABA-B receptor activation, promote nerve regeneration and likely ameliorate neuropathic pain.

## 1. Introduction

Neuropathic pain is primed by lesions to the somatosensory nervous system. It is characterized by a reduced nociceptive threshold and sometimes by spontaneous pain in the absence of stimuli, so that normally innocuous stimuli can produce pain. As a consequence, neuropathic pain may be partially associated with a state of tactile hypersensitivity (allodynia) and hyperalgesia [1]. These altered sensory features may be reproduced in rodent experimental models by a unilateral partial injury of the sciatic nerve. The “partial sciatic ligation” (PSL) shows many of the typical symptoms of the complex regional pain syndrome- (CRPS-) II, a human chronic pain condition associated with nerve injuries, including the rapid onset of allodynia [2]. In rodents, the behavioural hypersensitivity can be measured as a reduction in the threshold of sensory stimulation required for limb withdrawal behaviour. However, very few studies correlated the PSL-induced neuropathic pain with nerve morphology. Recently, studies performed with the PSL model have proposed new drug candidates and therapeutic targets to reduce neuropathic pain [3–7]. However, current pharmacotherapies for neuropathic pain are still unsatisfactory.

Although the involvement of the somatosensory system is associated with a wide range of neuropathic pain conditions, ranging from peripheral neuropathy to central post-stroke pain, neuropathic pain may be the consequence of a painful peripheral nerve injury [8]. In the last decade, given the increasing knowledge of the mechanisms regulating degeneration/regeneration of peripheral nerves several approaches to promote nerve regeneration have been addressed. The administration of neurotrophins [9–11], extracellular matrix molecules [12, 13], and cyclic adenosine monophosphate (cAMP) modulators [14] or the application of electrical stimulation [15, 16] has been proposed. Unfortunately, none of these methods was introduced in clinic, and currently available therapies are mainly addressed to the control of painful symptoms rather than to treat nerve degeneration and/or to promote regeneration. New strategies that simultaneously control nerve regeneration and neuropathic pain are needed.

The first working hypothesis that we tried to validate was to assess whether the PSL induces changes (e.g., in allodynia) that may correlate with alterations in nerve morphology and/or in myelin proteins expression.

The gamma-aminobutyric acid (GABA) signalling system has been proposed as a target of possible therapies addressed to promote the recovery from neuropathic pain [17–19]. GABA-B receptors are present in the neuronal compartment of the spinal cord white matter [20, 21] and are highly expressed in the laminae I–IV of the dorsal horn [22–25]. At these sites, GABA-B receptors colocalize with glutamic acid decarboxylase (GAD) enzyme and primary afferent fibers [23, 26, 27]. GABA-B receptor isoforms were also found in the rat dorsal root ganglia (DRG), in peripheral axons, in autonomic nerve terminals, and in pig nodose ganglion cells [22, 28–32]. Mice lacking functional GABA-B receptors exhibit pronounced hyperalgesia in a number of acute pain tests, corroborating the hypothesis of a role for GABA-B receptors in nociception [33, 34]. Very recently, Schwann cells were shown to express different isoforms of GABA-B receptor, such as −1a, −1b, and −2, which can participate in the control of the Schwann cell development and in the myelination process [35, 36]. The peripheral GABA-B receptors in Schwann cells may be involved in the regulation of the sensory and nociceptive functions [37, 38].

A number of clinical studies suggested that GABA-B agonists might be useful for the treatment of neuropathic pain. For example, the GABA-B agonist baclofen showed antinociceptive effects in the postherpetic neuralgia, in diabetic neuropathy, and in migraine [39–42]. Following systemic, supraspinal, and spinal administration, baclofen proved antinociceptive in animal models of chronic pain [43–47].

Therefore, we hypothesized that the modulation of the peripheral GABA-B receptors in nerves may contribute to ameliorate neuropathic pain via promotion of nerve regeneration. To test this working hypothesis, here we evaluated the putative therapeutic effects of two GABA-B ligands (the agonist baclofen and the antagonist CGP56433) on some behavioural, biochemical, and morphological parameters of peripheral nerves. The rationale to use either a GABA-B agonist or an antagonist was to test whether the GABA-B-mediated action in the PNS may be the consequence of a receptor stimulation or inhibition.

## 2. Materials and Methods

### 2.1. Animals and Surgery

In this study male Sprague-Dawley rats were used (Charles River, Calco, Italy). All the animals were anesthetized by an intraperitoneal (i.p.) injection of a mixed solution of ketamine (80 mg/Kg) and xylazine (5 mg/Kg). The experimental model of PSL has been achieved following the method of Seltzer et al. [2]. Briefly, an incision was made through skin and muscle of the right hind limb. The nerve was then exposed and, for the experimental groups, ligated with a node made using a suture (Vicryl 3-0 2), which crossed the nerve at half or 1/3 of its diameter. The lesion was done 1 cm proximally to the forking of the sciatic nerve into the peroneal and tibial branches. Seven days after surgery, animals were terminated by anesthetic overdose and the nerves collected at −80°C. All the experiments were performed according to the Animal Research Committee of our Institution and in compliance with the policy on the use of animals approved by the European Community Council Directive (2010/63/EC). Our study was approved by the Animal Care and Use Committee.

### 2.2. Pharmacological Treatments

In each experiment, 4 rats were used as sham operated animals (control group), while 16 rats underwent PSL. The PSL rats were then divided into 4 experimental groups for treatments: (1) PSL + vehicle (saline solution); (2) PSL + baclofen (10 mg/kg); (3) PSL + CGP56433 (3 mg/kg); (4) PSL + baclofen + CGP56433. The pharmacological treatments started the day of PSL surgery (day 0) and lasted 7 days, consisting in the administration of i.p. injection twice a day. Experiments were done at least 3 times. All GABA-B compounds were provided by K. Kaupmann, Novartis Pharma AG, Basel, Switzerland.

### 2.3. Mechanical Allodynia and Thermal Hyperalgesia

Animals were accustomed to test cages before behavioural tests. Responses to mechanical and thermal stimuli were performed on the ipsilateral hind paws of sham and PSL rats at different time points (i.e., 1 day and 7 days postsurgery). Mechanical allodynia was assessed using the dynamic plantar aesthesiometer (Ugo Basile, Comerio, Italy). Rats were placed in the test cage with a wire mesh floor and a rigid tip of a Von Frey-filament (punctate stimulus) was applied in the middle of the skin of the hind paw. The filament exerted an increasing force, up to 45 g in 35 sec. We measured the withdrawal threshold, expressed in grams (g), until each animal removed its paw. Thermal hypersensitivity was tested according to the Hargreaves procedure [48] using the Basile plantar test apparatus (Ugo Basile). A constant intensity radiant heat source (beam diameter 0.5 cm and intensity 40 I.R.) was aimed at the midplantar area of the hind paw. We recorded [in seconds (s)] the time interval (named as latency) from the heat source activation, until each animal withdrew its paw. The thresholds were measured repeatedly, to each time point (1 day and 7 days postsurgery), and the values are the mean of 4 evaluations of each animal ± SEM.

### 2.4. RNAse Protection Assay (RPA)

Total RNA was isolated from sciatic nerves by phenol-chloroform extraction and 5 *μ*g of each sample were processed as described [49]. Briefly, the samples were dissolved in 20 *μ*L of hybridization solution containing [^32^P]-labeled cRNA probes, 250,000 counts per minute (CPM) for glycoprotein P0 (P0) and peripheral myelin protein of 22 kDa (PMP22) and 50,000 CPM for 18S, and incubated at 45°C overnight. The following day, the samples were incubated 30 min at 30°C with 200 *μ*L of digestion buffer containing 1 *μ*g/*μ*L RNase A and 20 U/*μ*L RNase T1 (Sigma-Aldrich, Milan, Italy) then treated for 15 min at 37°C with 10 *μ*g of proteinase K and sodium dodecyl sulfate (SDS) 20%. After phenol-chloroform extraction, the samples were separated on a 5% polyacrylamide gel, under denaturing conditions (7 mol/L urea). The protected fragments were visualized by autoradiography. The levels of P0 and PMP22 and 18S rRNA were calculated by measuring the peak densitometric area with Image J software (NIH, USA) and data were normalized versus 18S rRNA. In order to ensure that the autoradiographic bands were in the linear range of intensity, different exposure times were used. The mean values of the controls from different experiments were within 10% of each other.

### 2.5. Light Microscopy (LM) and Morphological Analysis

Rats were perfused transcardially, under deep anesthesia, with a solution containing 2% PFA and 2% glutaraldehyde in 0.1 M sodium cacodylate buffer (Sigma-Aldrich) pH 7.3. After fixation sciatic nerves were removed and immersed in the same fixative solution overnight at 4°C. The specimens were then postfixed in 2% OsO_4_ (Sigma-Aldrich), dehydrated, and embedded in Epon-Araldite (Sigma-Aldrich). Semithin (0.5 *μ*m) transverse sections were stained with toluidine blue and analyzed with a Axioskop200 microscope (Zeiss, Gottingen, Germany), at final 1500x magnification. To assess morphological alterations of peripheral nervous system (PNS), qualitative and quantitative analyses were performed. Usually, 5 sections for each animal nerve were analysed. At least 25 fields for each section, corresponding to 25% of the total nerve area, were acquired [50]. The number of myelinated fibers was counted, normalized per field (in *μ*m^2^), and expressed as mean ± SEM of data.

### 2.6. Electron Microscopy (EM)

Ultra-thin sections (60–90 nm) for ultrastructural observations were obtained from each sciatic nerve used for the light microscopy. Sections were collected on formvar film coated grids with a single hole, counterstained with lead citrate, and examined with a Zeiss EM10 electron microscope.

### 2.7. Immunofluorescence and Confocal Laser Scanning Microscopy (CLSM)

Sciatic nerves were fixed in 4% PFA in phosphate buffered saline (PBS; Sigma-Aldrich) and 10 *μ*m transverse frozen sections were cut. Nerve sections were incubated 30 min at room temperature in presence of Fluoromyelin^TM^ red fluorescent myelin stain (1 : 200, Life Technologies Italia, Monza, Italy). Slides were then incubated overnight at 4°C in PBS 0.25% bovine serum albumin (Sigma Aldrich), 0.1% Triton X-100, and the mouse monoclonal anti-CD68 primary antibody (1 : 200 AbCam, Cambridge, UK). The following day slides were washed two times and incubated 2 h with the specific goat anti-mouse FITC-antibody (1 : 200; Sigma-Aldrich). After washing, nuclei were stained with 4′,6-diamidino-2-phenylindole (DAPI) and slides were mounted using Vectashield^TM^ (Vector Laboratories, Burlingame, CA, USA). Controls for specificity included a lack of primary antibody. Confocal microscopy was carried out using a Zeiss LSM 510 microscope and Image Pro-Plus 6.0 software (MediaCybernetics, Bethesda, MA, USA).

### 2.8. Walking Test

The walking footprint test was done on all rats of each experimental group, at the end of pharmacological treatments. Rats with their hind feet stained with black ink were placed on a 100 cm long gangway, which yielded at least 10 footprints per rat. Rats did not undergo any training before the test. Footprints were acquired and analysed with the Footprint 1.22 program [51]. We measured the image area touched by a single step (in cm^2^), the toe 1–5 and 2–4 spreads (in cm), the length of the foot step (in cm), the stride width and the stride length (in cm). Data of each parameter are mean of 8–10 footprints of each animal ± SEM.

### 2.9. Data Analysis and Statistics

Data were statistically evaluated by using GraphPad Prism 4.0 software (San Diego, USA) and Systat 12 software (Systat Inc., Chicago, USA). Significance was determined by one-way Anova analysis for multiple comparison using Tukey's post hoc test for morphometric, gait, and myelin proteins expression analysis and by two-way Anova analysis using Bonferroni's post hoc test for the analysis of nociception. Quantitative data include 95% confidence intervals. *P* values < 0.05 were considered significant.

## 3. Results

### 3.1. Assessment of the PSL Experimental Model

**Figure 1:**
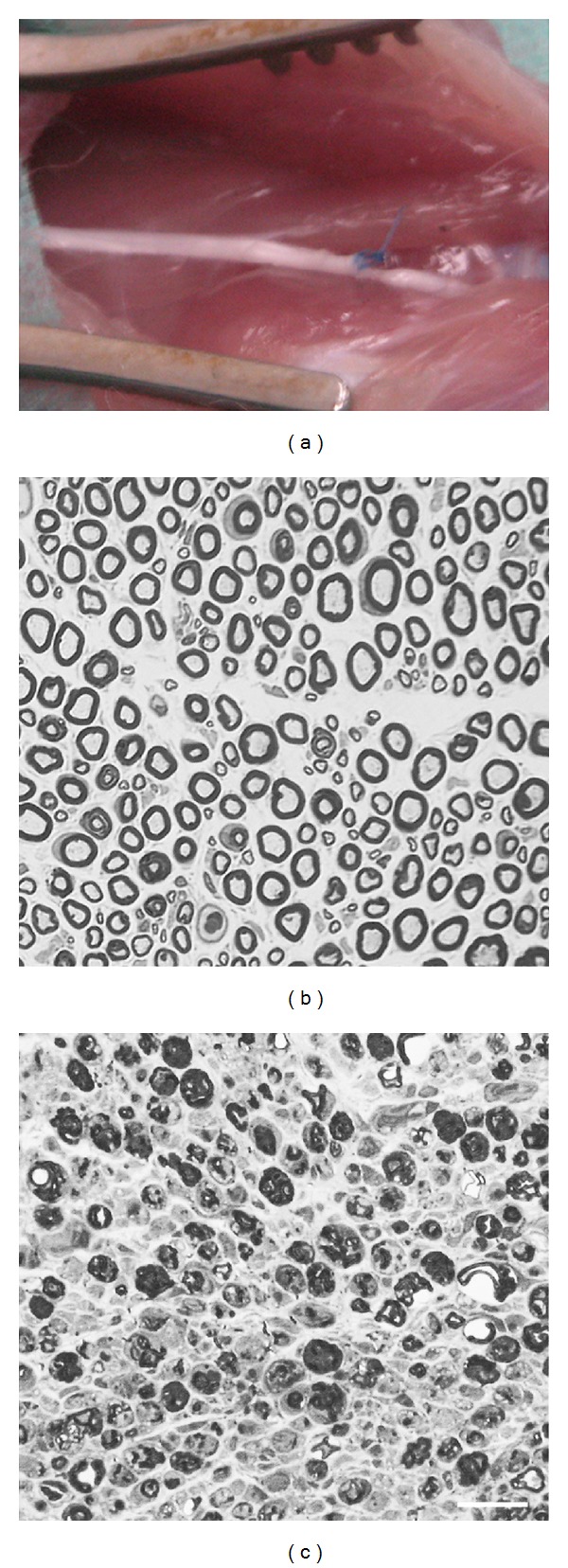
PSL experimental model induces neurodegeneration of peripheral nerves. (a) Image of the partial ligation of the right sciatic nerve. (b) Toluidine blue stained semithin cross-section of sham sciatic nerve in which normal myelinated fibers are appreciable. (c) Toluidine blue stained semithin cross-section of a ligated sciatic nerve showing signs of massive neurodegeneration. Scale bar 20 *μ*m.

The right sciatic nerve was exposed and then tightened with a suture which crossed the nerve approximately at 1/3 of its diameter (Figure 1(a)). No signs of massive inflammatory reaction or serum infiltrate were found (data not shown). Although PSL has been extensively studied as a behavioural model of neuropathic pain, few proofs of its capability to induce morphological changes are available. The nerve degeneration was analysed 7 days after ligation, a time point presenting the typical signs of axonal atrophy and myelin degradation (Figure 1(c)). The sham operated animals (controls) presented normal nerve morphology (Figure 1(b)). Considering that the PSL-induced denervation is equal for axons of all sizes [52], we counted the number of myelinated fibers per field as an indirect index of nerve integrity (Table 1). They resulted normal in sham operated rats (18.04 ± 1.12 fibers; 95% CI: 15.81–20.26), while were significantly decreased in PSL rats (2.52 ± 0.12 fibers; 95% CI: 2.28–2.76; *P* = 0.000017 versus sham operated).

### 3.2. In PSL Rats the Mechanical Allodynia Is Counteracted by GABA-B Ligands

**Figure 2:**
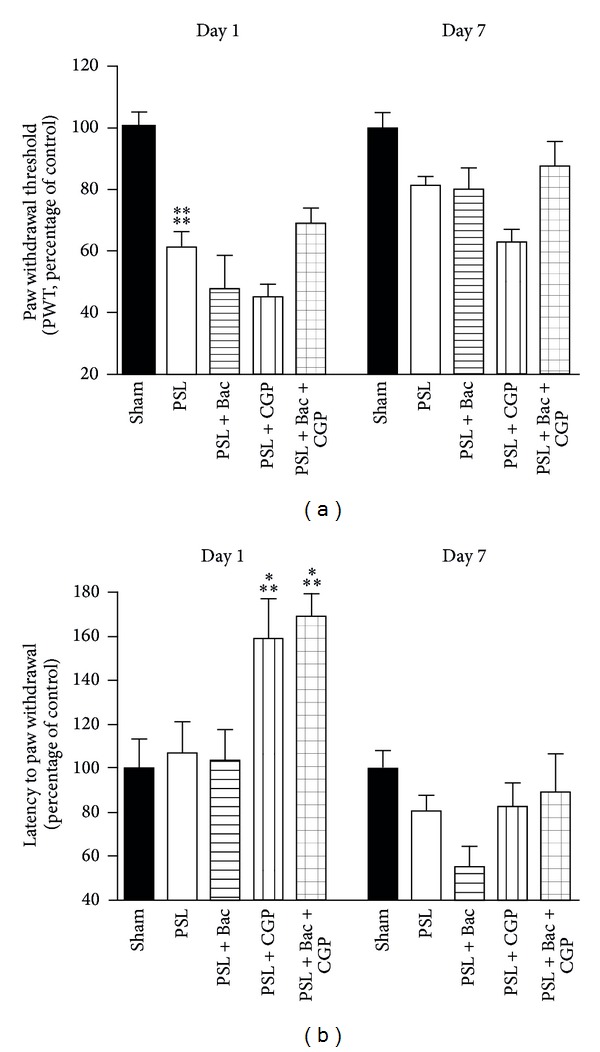
Assessment of allodynia and hyperalgesia after GABA-B ligands treatment. (a) Histograms of the paw withdrawal threshold (PWT) of rats to innocuous stimulation with a filament, at 1 and 7 days postsurgery. Data are expressed as percentage versus controls (sham operated). (b) Histograms of thermal nociception of rats with a filament, at 1 and 7 days postsurgery. Latencies to paw withdrawal (in sec) were assessed at infrared intensity 20, and data are expressed as percentage versus controls (sham operated). Statistic with two-way Anova and Bonferroni's post hoc test. ***P < 0.001, ****P < 0.0001.

We expected PSL to induce allodynia and hyperalgesia in the injured rats. To confirm this hypothesis we evaluated the nociceptive responses to mechanical and thermal stimuli, after PSL and in the presence of GABA-B ligands. As shown in Figure 2(a), the paw withdrawal threshold (PWT) of PSL rats to mechanical stimuli decreased significantly 1 day after nerve injury (diff. −13.56 versus sham operated; 95% CI: −23.49/−3.63; *P* = 0.000099) and lightly increased at 7 days (diff. −5.57 versus sham operated; 95% CI: −17.76/6.61; *P* = 0.187689), despite being still lower of the sham operated animals at 7 days postinjury. This indicates an allodynic state. The cotreatment for 7 days with baclofen (10 mg/kg) plus CGP56433 (3 mg/kg) caused a significant recovery of the response to mechanical stimuli to the levels observed in the sham animals (diff. −3.77 versus sham operated; 95% CI: −14.46/6.91; *P* = 0.308831), suggesting that the cotreatment counteracts allodynia. At 7 days, neither baclofen nor CGP56433 alone proved able to induce any recovery to the levels of sham animals. Then, the latency to paw withdrawal (in sec) to acute thermal stimuli was assessed. As shown in Figure 2(b), at 7 days postinjury the paw withdrawal was lower in PSL rats in comparison to the sham operated rats (diff. −5.66 versus sham; 95% CI: −16.12/4.81; *P* = 0.119657). This suggests a hyperalgesic state. As for the mechanical allodynia, the treatment with baclofen plus CGP56433 was effective. Indeed, it induced a significant hypoalgesia 1 day postsurgery (diff. 13.86 versus sham; 95% CI: 2.76/24.96; *P* = 0.000361) and tended to recover the levels of sham operated animals at 7 days (diff. −3.15 versus sham; 95% CI: −15.29/9.01; *P* = 0.455421). Also CGP56433 alone revealed a similar significant response to a nociceptive thermal stimuli at 1 day (diff. 11.70 versus sham operated; 95% CI: 1.87/21.54; *P* = 0.000675), but at 7 days it showed a slighter recovery, tending to the levels of sham animals (diff. −4.91 versus sham operated; 95% CI: −15.02/5.22; *P* = 0.163287).

### 3.3. GABA-B Ligands Improve the Peripheral Nerve Morphology

**Figure 3:**
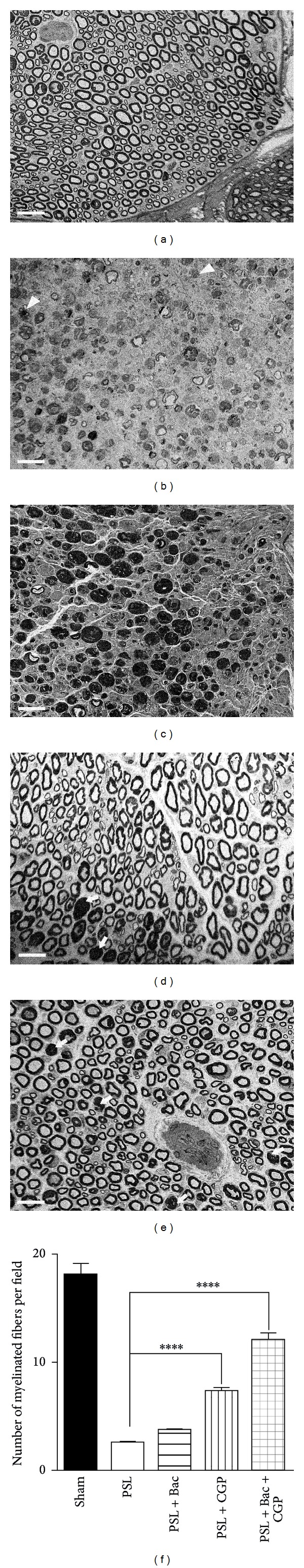
Morphological alterations of peripheral nerves are recovered after GABA-B ligands treatment. Toluidine blue stained semithin cross-sections of (a) sham operated rat. Evidence of normal structure of the nerve. (b) PSL rat in which a loss of myelinated fibers is evident. The nerve fiber structure appears degenerated and macrophages (indicated by arrowheads) are observed. (c) PSL rat treated with baclofen 10 mg/kg. Nerve appears still degenerated with a significant loss of myelinated fibers. (d) PSL rat treated with CGP56433 3 mg/kg. Nerve shows normal myelinated fibers, although some areas still contain degenerated axons (arrows). (e) PSL rat treated with baclofen + CGP56433. Nerve shows a regeneration pattern with several normal myelinated fibers and very few degenerated axons (arrows). Scale bar 20 *μ*m. (f) Quantitative morphometric analysis of the number of myelinated fibers. All data are expressed as means ± SEM. Statistic with one-way Anova and Tuckey's post hoc test. ****P < 0.0001.

To test the hypothesis whether the nociceptive responses may correlate with morphological changes, the structure of degenerated PSL nerves was analysed by light microscopy. At least 5 semithin transverse sections (0.5 *μ*m) of each animal were examined along the degenerated/regenerated nerves, covering all the distance from the upper to the lower parts of the suture crossing the nerve. As shown in Figure 3(c), the 7-day treatment with baclofen (10 mg/kg i.p.) did not change the structure, which resembled that of PSL rats, with degenerated nerve fibers and some infiltrated macrophages (Figure 3(b)). Nonetheless the CGP56433 treatment (3 mg/kg, i.p.) produced a full amelioration of the nerve structure (Figure 3(d)). Similarly the administration of baclofen (10 mg/kg) plus CGP56433 (3 mg/kg) improved the nerve morphology (Figure 3(e)), which qualitatively resembles that of sham operated rats (Figure 3(a)). To validate these qualitative observations, we quantified the number of myelinated fibers, as an index of nerve integrity, at 7 days posttreatment (Table 1). As shown in Table 1 and Figure 3(f), sham operated rats showed normal number of myelinated fibers (18.04 ± 1.12; 95% CI: 15.81–20.26) per field, while the PSL rats showed a significant reduction (2.52 ± 0.12; 95% CI: 2.28–2.76; *P* = 0.000017 versus sham operated). However, the number of myelinated fibers progressively increased, depending on the treatment. Indeed, baclofen did not produce any significant increase (3.63 ± 0.23; 95% CI: 3.18–4.07; *P* = 0.438372 versus PSL), while CGP56433 significantly raised the number of myelinated fibers (7.31 ± 0.36; 95% CI: 6.61–8.01; *P* = 0.000017 versus PSL). However only the simultaneous treatment with baclofen and CGP56433 was able to determine a significant recovery of the myelinated fibers to sham operated levels (12.02 ± 0.71; 95% CI: 10.62–13.43; *P* = 0.000017 versus PSL).

**Figure 4:**
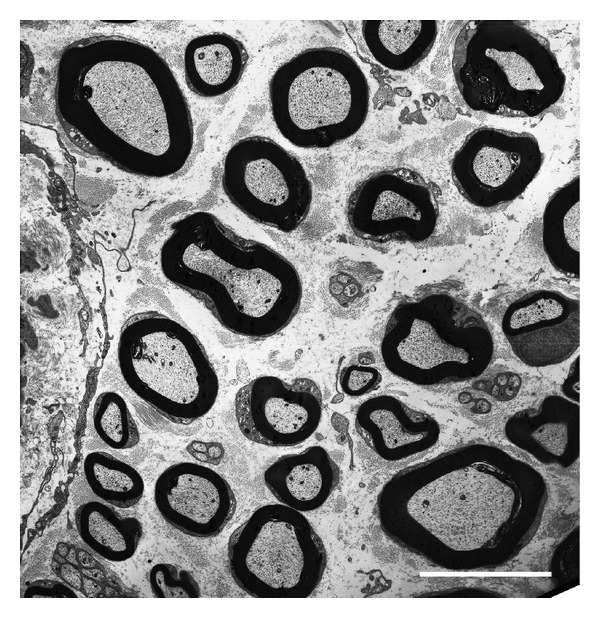
Electron micrograph picture of ultra-thin cross-section of PSL sciatic nerve after treatment with baclofen + CGP56433. Normal myelinated large fibers and small thinly myelinated regenerating fibers are shown. Scale bar 10 *μ*m.

The recovery of a normal anatomical structure of the nerve after baclofen plus CGP56433 treatment was confirmed also by the EM analysis. Indeed, we observed normal myelin laminae and Schwann cells, with several small fibers, thinly myelinated indicative of starting of nerve regeneration (Figure 4).

### 3.4. The Myelin Proteins Are Changed by the GABA-B Ligands

**Figure 5:**
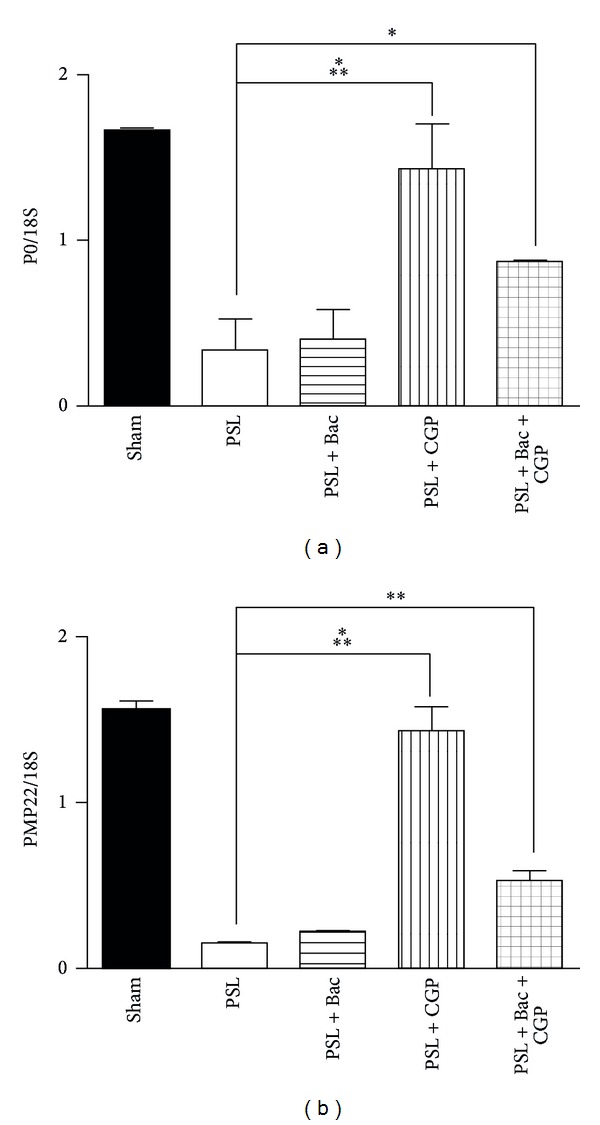
The gene expression of glycoprotein P0 and PMP22 is modulated by GABA-B ligands. (a) Histograms of P0 mRNA levels measured by RNAse protection assay. (b) Histograms of PMP22 mRNA levels measured by RNAse protection assay. For both proteins data were normalized versus 18S rRNA levels and expressed as mean ± SEM. Statistic with one-way Anova and Tuckey's post hoc test. *P < 0.05, **P < 0.01, ***P < 0.001.

To assess whether the morphologic changes may be due to a direct effect on the myelinating Schwann cells, we then analysed the expression of the most important myelin proteins, P0 and PMP22. P0 is a glycoprotein accounting for over half of the total proteins in PNS myelin, while PMP22 represents 2–5% of the total amount of the peripheral myelin proteins [53]. They are essential constituents required for stabilizing compact myelin, in fact their quantitative changes may alter Schwann cell development, growth, and differentiation [54]. For instance, a gene deletion or a gain of function of these proteins can cause congenital neuropathies such as the Charcot-Marie-Tooth (CMT) disease [54]. As shown in Figures 5(a) and 5(b) the gene expression of P0 (0.34 ± 0.08; 95% CI: 0.14–0.53; *P* = 0.000137 versus sham operated) and PMP22 (0.14 ± 0.02; 95% CI: 0.08–0.19; *P* = 0.000128 versus sham operated) was significantly decreased in PSL rats. The 7-day treatment with CGP56433 (3 mg/kg) significantly restored the P0 (1.43 ± 0.27; 95% CI: 0.73–2.13; *P* = 0.000146 versus PSL) and PMP22 (1.44 ± 0.14; 95% CI: 1.07–1.80; *P* = 0.000128 versus PSL) mRNAs to the levels of sham operated animals. Baclofen (10 mg/kg) alone did not change the gene expression levels of both P0 and PMP22 proteins. Surprisingly, as shown in Figures 5(a) and 5(b), baclofen plus CGP56433 showed a partial but significant increase in the mRNA levels of both myelin proteins: P0 (0.86 ± 0.01; 95% CI: 0.85–0.87; *P* = 0.028372 versus PSL) and PMP22 (0.53 ± 0.06; 95% CI: 0.38–0.68; *P* = 0.008942 versus PSL).

### 3.5. Functional Assessment of the Nerve Regeneration Induced by the GABA-B Ligands

**Figure 6:**
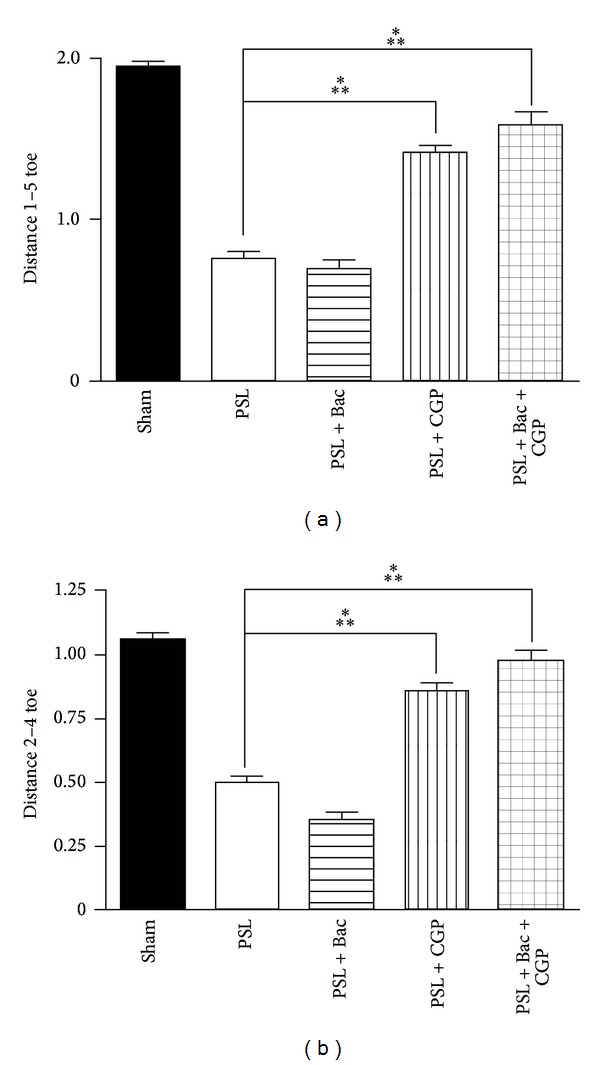
Assessment of locomotor coordination and gait analysis after GABA-B ligands treatment. (a) Histograms of the analysis of the distance between toes 1–5. (b) Histograms of the analysis of the distance between toes 2–4. Data are expressed as mean ± SEM. Statistic with one-way Anova and Tuckey's post hoc test. ***P < 0.001.

The functional recovery of nerves in PSL rats was then assessed by evaluating the motor coordination and synchrony with the gait analysis. The rats were allowed to walk in a standard corridor, leaving a trail of footprints, which were analysed measuring the following parameters: stride length and width, step length and area, distance between 1–5 and 2–4 toes. In the PSL rats at 7-day postsurgery most of the parameters were significantly altered, if compared to the sham operated animals (see data in Table 2). These results confirmed that the PSL injury induces a functional motor deficit. Baclofen alone did not show significant improvements in toes distances 1–5 and 2–4 and in length of foot step, versus PSL (see data in Table 2). Furthermore, baclofen worsened some parameters (Table 2), for instance, the distances of toes 2–4, that was significantly decreased in PSL treated with baclofen (0.35 ± 0.03; 95% CI: 0.29–0.41; *P* = 0.000131 versus sham). However other parameters, such as stride width, length, and foot step area, were significantly increased after baclofen treatment (see data in Table 2). The pharmacological treatment with CGP56433 or cotreatment baclofen plus CGP56433 reversed almost all the parameters affected by PSL (Table 2). The CGP56433 treatment significantly increased the 1–5 (1.42 ± 0.05; 95% CI: 1.32–1.51; *P* = 0.00116 versus PSL) and 2–4 (1.58 ± 0.08; 95% CI: 1.40–1.76; *P* = 0.000135 versus PSL) toes distances, to the levels comparable to sham operated rats (Figure 6). The most effective treatment was baclofen plus CGP56433 that significantly recovered the 1–5 (0.35 ± 0.03; 95% CI: 0.29–0.41; *P* = 0.000116 versus PSL) and 2–4 (0.98 ± 0.04; 95% CI: 0.90–1.06; *P* = 0.000131 versus PSL) toes distances, to the levels of sham operated rats (Figure 6).

### 3.6. GABA-B Ligands Reduce the Nerve Inflammatory Cells

**Figure 7:**
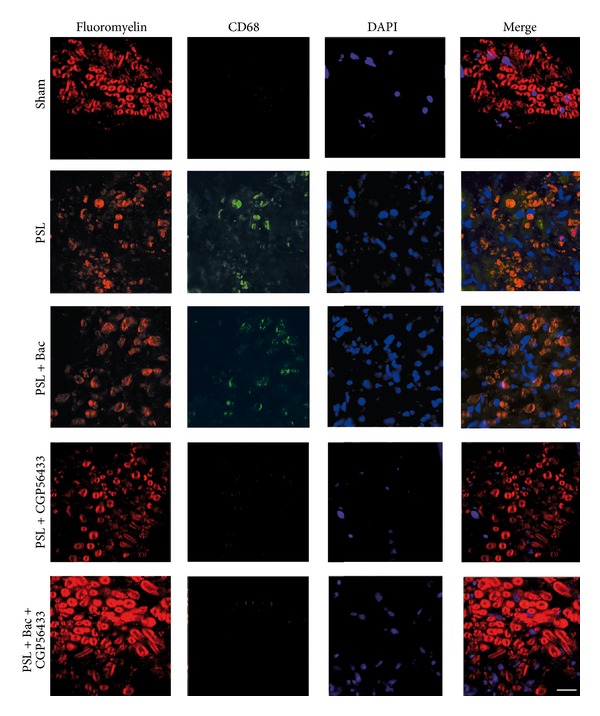
Neuroimmune response in peripheral nerves is modulated by GABA-B ligands. Immunofluorescence images of coronal sections of sciatic nerves of sham and PSL rats treated with baclofen 10 mg/kg, CGP56433 3 mg/kg, and baclofen plus CGP56433. Myelin structures are evidenced in red with Fluoromyelin. Macrophages specific marker CD68 is in green. Nuclei are stained in blue with DAPI. Scale bar 20 *μ*m.

We evaluated the recruitment of activated macrophages, assessing the immunopositivity for CD68 as an index of nerve inflammation. The transverse sections of sciatic nerves showed the typical normal myelin structure, evidenced in red with Fluoromyelin^TM^ staining, in sham and CGP56433 treated rats (Figure 7). As expected, the myelin rings were altered in PSL and baclofen treated animals, confirming the morphologic results. The absence of CD68 immunopositivity excluded any macrophage infiltrate in sham operated animals. By contrast, in PSL and in PSL plus baclofen rats several macrophages infiltrated the nerve, indicating a general increase in neuroinflammation (CD68 in green). In particular the merged images (in yellow) revealed a CD68 positivity in the cells infiltrating the myelin structure (Figure 7). Indeed, the infiltrated macrophages totally disappeared after treatment with CGP56433 or with baclofen plus CGP56433 (Figure 7), demonstrating that only the antagonist CGP56433 proved able to counteract the macrophagic recruitment in the nerve, regardless of the presence of baclofen.

## 4. Discussion

The identification of new treatments for nerve regeneration and chronic neuropathic pain is currently a challenge for clinical neurologists. Improvements in therapy rely on the complete comprehension of the mechanism inducing these pathologies. This will contribute to change the future therapy of pain from a symptomatic towards a molecular approach. By using the PSL model of nerve injury, we observed the onset of tactile hypersensitivity (i.e., allodynia) beside an alteration of the nerve morphology and myelin proteins expression.

PSL is an experimental model that mimics the complex regional pain syndrome named CRPS-II, including the complex combination of rapid onset allodynia and hyperalgesia [1, 2]. However, the morphological features of injured nerves and the expressions of the two most important peripheral myelin proteins, P0 and PMP22 [53], were poorly investigated. We observed a severe degeneration in the nerve structure with loss of large myelinated fibers, nerve demyelination, and phagocytosis. The P0 and PMP22 expression was strongly decreased, confirming a massive degenerative process affecting the Schwann cells-myelin forming compartment of the peripheral nerves. According to the literature [52], we expected that the nerve degeneration would be equal for axons of all sizes and may affect either the myelinated (type Abeta/Adelta) or the unmyelinated (type C, mostly nociceptors) fibers.

Additionally, we evaluated the putative therapeutic effects of two GABA-B ligands on the altered behavioural, biochemical, and morphological parameters of the PSL model. The choice to use baclofen, as a GABA-B ligand, relies on our previous findings demonstrating the involvement of GABA-B receptors in PNS. GABA-B receptor is important for the control of Schwann cell biology, for the process of myelination and for the peripheral nociception [36, 37, 55, 56]. Despite the role of GABAergic system in neuropathic pain, however, GABA-B receptor agonists are rarely used in clinic to treat neuropathic pain mainly because of their narrow therapeutic window. Baclofen showed antiallodynic and antinociceptive actions in chronic pain models in rats, such as the PSL [57, 58]. Baclofen was also used in a limited number of clinical trials to treat some types of neuropathic pain, including the use of spinally administered baclofen to enhance the spinal stimulation effects [39, 42, 59]. However, its use was limited because of its sedative properties and the rapid development of tolerance. Indeed, CGP56433 is a high-affinity antagonist of GABA-B receptor, active at nanomolar concentration in numerous* in vitro* and* in vivo* studies [60, 61]. CGP56433 was not previously used for regenerative purposes of the PNS, so that our results are the first to determine the potentiality of GABA-B ligands for peripheral nerve repair and for the concomitant control of neuropathic pain.

Surprisingly, in our studies the agonist baclofen and the antagonist CGP56433 exerted a synergic effect. This additive phenomenon in the nervous system is not common, although a GABA-B ligand synergic effect on brain stimulation rewards was described [62]. The GABA-B agonist CGP44532 and the antagonist CGP56433 induced a reward decrement when administered separately, while their coadministration induced an additive effect on threshold, rather than blocking the agonist-induced threshold elevations [62]. However, part of the effects achieved with CGP56433 treatment alone (for instance the nociceptive responses, the number of myelinated fibers, and the gait) may result from the blocking of GABA-B receptor activation by endogenous GABA. This is compatible with the capacity of peripheral nerves (i.e., the Schwann cells) to synthesize and release GABA [63]. Schwann cells, indeed, resulted to be particularly responsive to CGP56433, revealing a specific effect mediated by the GABA-B receptors present on their cell surfaces. Baclofen decreases the proliferation and the myelin proteins expression in Schwann cells [36]. As expected, the GABA-B antagonist significantly upregulated the levels of the myelin proteins P0 and PMP22.

Considering the possible involvement of Schwann cells in neuropathic pain [64], our findings suggest that the synergic effects exerted by baclofen plus CGP56433 may be the result of a double simultaneous action of CGP56433, through the myelin-forming component and by baclofen, likely through the neuronal (central and/or peripheral) compartment. Indeed, given the synergic effects on locomotors activities, the activation of mixed central and peripheral targets by those molecules should not be excluded. However, very recent findings supported the hypothesis that GABA-B receptors may also control the nonmyelinating Schwann cells committed to ensheath the nociceptive fibers, strengthening a GABA-B role in the regulation of Schwann cell fate and nociceptive pathways in the PNS [37, 38].

PSL induces early signs of damage of the motor compartment. They include loss of normal spread between toes (e.g., 1–5 and 2–4 toes), loss of ventroflexion of toes, and consequent increase of length print [65]. Our gait analysis is in agreement with these events, confirming a PSL-induced damage of the nerve peripheral motor compartment and a CGP56433/baclofen significant recovery to normal gait. Interestingly, CGP56433 alone partially recovered the 1–5 and 2–4 toes distances, suggesting that these effects may be ascribed to the antagonism of the GABA-B mediated tonic control of motor coordination.

In the PNS, macrophages are committed to neuroimmune surveillance and they intervene during the Wallerian degeneration following axonal injury [66]. Macrophages concur to the onset of pain hyperactivity; thus a decrease in their recruitment is a desirable process [64]. The lack of their recruitment that we observed following CGP56433 and CGP56433-baclofen treatments is in agreement with a protection against neuroimmune-induced neuropathic pain, revealing that only the GABA-B antagonist is active. This is not surprising, since GABA-B receptors were found in macrophages [67].

Although we believe that most of the effects observed are due to a direct action of drugs on peripheral components, the involvement of central pathways should not be excluded. It is indeed reported that central mechanisms of GABA ligands, administered intrathecally, equally affect the behavioural signs of neuropathy [46, 68]. Therefore, the role of GABAergic system in pain appears to be quite complicated, since the transmitter may have both pro- and antinociceptive effects [69–71], and the route of administration may discriminate in this sense. The possibility to administer GABA-B ligands* in situ* by a drug delivery system, close to the nerve injury site, is an important issue to minimize the central effects, which deserves further investigations.

## 5. Conclusions

In this paper we tried to shed light on the molecular mechanisms associating GABA-B receptors, nerve degeneration/regeneration, and peripheral nociception. We hypothesised that GABA-B mediated effects arise from direct and indirect effect on the axonal and Schwann cell compartments, respectively. Although an activation of mixed central and peripheral effects should not be excluded, our results suggest that the simultaneous treatment with baclofen and GCP56433 exerts a peripheral synergic effect in promoting nerve regeneration after injury and likely ameliorates the neuropathic pain.

## Figures and Tables

**Table 1:** Stereologic evaluation of number of myelinated fibers in peripheral nerves of sham, PSL, and PSL-treated rats.

		Number of myelinated fibers per field	
Sham	18.04	±1.12	
PSL	2.52	±0.12	*P* < 0.0001 versus sham
PSL + Baclofen	3.63	±0.23	
PSL + CGP56433	7.31	±0.36	*P* < 0.0001 versus PSL
PSL + Baclofen + CGP56433	12.02	±0.71	*P* < 0.0001 versus PSL

Statistic with one-way Anova and Tuckey's post hoc test.

**Table 2:** Footprint analysis of most important parameters of locomotor activity in sham, PSL, and PSL-treated rats.

	Distance 1–5 toe		Distance 2–4 toe		Length foot step		Stride width		Stride length		Area touched	
Sham	1.95	±0.03	1.06	±0.03	3.21	±0.09	3.64	±0.06	11.46	±0.49	1.79	±0.08

PSL	0.76	±0.04	0.50	±0.03	4.39	±0.13	4.59	±0.11	13.32	±0.54	1.90	±0.17
	*P* = 0.000116		*P* = 0.000131		*P* = 0.000116		*P* = 0.000119		*P* = 0.404063		*P* = 0.968606	

PSL + baclofen	0.70	±0.05	0.35	±0.03	4.10	±0.18	3.85	±0.15	9.60	±0.71	1.06	±0.14
	*P* = 0.921960		*P* = 0.547398		*P* = 0.648819		*P* = 0.002619		*P* = 0.018476		*P* = 0.000914	

PSL + CGP56433	1.42	±0.05	0.86	±0.03	2.61	±0.12	3.28	±0.11	9.29	±0.64	1.13	±0.20
	*P* = 0.000116		*P* = 0.000135		*P* = 0.000113		*P* = 0.000117		*P* = 0.018430		*P* = 0.005125	

PSL + baclofen + CGP56433	1.58	±0.09	0.98	±0.04	3.51	±0.12	3.63	±0.15	10.16	±0.93	1.51	±0.11
	*P* = 0.000116		*P* = 0.000131		*P* = 0.000466		*P* = 0.000129		*P* = 0.056044		*P* = 0.289398	

Data are expressed as mean (in cm) ± SEM. Statistic with one-way Anova and Tuckey's post hoc test. Significance for PSL was calculated versus sham-operated, while significance for baclofen, CGP56433, and baclofen/CGP56433 groups was calculated versus PSL.
